# Determinants of Breastfeeding Practices Pre- and During the COVID-19 Pandemic: A Retrospective Cohort Study

**DOI:** 10.3390/healthcare13182379

**Published:** 2025-09-22

**Authors:** Maryam Sharfi, Nasreen Hejres, Asma Ali, Noor Aldoseri, Badreya Malalla, Maria Tolentino, Wafa Hamad Almegewly, Khulud Ahmad Rezq

**Affiliations:** 1 Postnatal Unit, King Hamad University Hospital, Royal Medical Kingdom of Bahrain Services, Al Sayh 24343, Bahrain; maryam.sharfi@rms.bh; 2 King Hamad University Hospital, Royal Medical Kingdom of Bahrain Services, Al Sayh 24343, Bahrain; nasreen.hejres@rms.bh (N.H.); asma.alisaleem@rms.bh (A.A.); noor.aldoseri@rms.bh (N.A.); badreya.mubarak@rms.bh (B.M.); 3 Nursing Quality, King Hamad University Hospital, Royal Medical Kingdom of Bahrain Services, Al Sayh 24343, Bahrain; maria.tolentino@rms.bh; 4 Department of Community and Psychiatric Mental Health Nursing, College of Nursing, Princess Nourah bint Abdulrahman University, P.O. Box 84428, Riyadh 11671, Saudi Arabia; 5 Community and Psychiatric Health Nursing Department, Faculty of Nursing, University of Tabuk, Tabuk 47512, Saudi Arabia; krizk@ut.edu.sa

**Keywords:** exclusive breastfeeding, maternal characteristics, COVID-19 impact, mode of delivery, hospitalization practices

## Abstract

**Background/Objectives:** Exclusive breastfeeding (EBF) during hospitalization is essential for infant health but remains suboptimal in many Gulf Cooperation Council (GCC) countries. Maternal factors and the COVID-19 pandemic may have influenced breastfeeding practices. Understanding these determinants is crucial for improving postnatal care. This study aimed to identify determinants of breastfeeding practices during hospitalization before and during the COVID-19 pandemic in Bahrain. **Methods:** This retrospective cohort study reviewed electronic records of 321 mothers and their newborns discharged between March 2019 and March 2021 from a larger pool of 4500 cases. A structured data collection form was used to capture maternal age, parity, nationality, mode of delivery, COVID-19 period of delivery (pre-pandemic vs. pandemic), breastfeeding method, and reasons for mixed feeding. Descriptive and inferential statistical analyses were conducted to identify associations between variables. **Results:** Multiparous mothers had significantly higher exclusive breastfeeding rates than primiparous mothers (*p* = 0.016). The mode of delivery showed a strong association with breastfeeding outcomes, with vaginal births linked to higher EBF rates (*p* < 0.01). A notable decline in EBF was observed during the COVID-19 pandemic period, from 40% pre-pandemic to 14% during the pandemic. **Conclusions:** Exclusive breastfeeding during hospitalization is significantly influenced by delivery method and maternal parity. Although the COVID-19 pandemic may have contributed to reduced EBF rates, further research is required to validate these trends. These findings emphasize the importance of supportive hospital policies, particularly for first-time and cesarean mothers, and stress the need to strengthen breastfeeding practices during public health emergencies.

## 1. Introduction

Breastfeeding is a vital public health strategy that provides optimal nutrition and protective immunity to infants while supporting maternal health. It is associated with reduced risks of infections, chronic diseases, and mortality among children and with lower incidences of breast and ovarian cancers in mothers [1]. Despite advances in healthcare, no artificial substitute replicates the immunological and nutritional benefits of human breast milk [2]. The World Health Organization (WHO) and the United Nations International Children’s Emergency Fund (UNICEF) recommend initiating breastfeeding within the first hour of life, maintaining EBF for the first six months, and continuing alongside complementary feeding for up to two years or more [3,4]. Evidence from a longitudinal perspective study indicates that children who were breastfed for six months or longer experienced reduced parent-reported illnesses at 6, 12, and 24 months of age and demonstrated a decreased likelihood of obesity by age three [5].

Despite growing global awareness of the benefits of EBF, significant regional disparities in breastfeeding practices continue to persist. UNICEF estimates that universal adoption of optimal breastfeeding could prevent approximately 823,000 child deaths and 20,000 maternal deaths annually, while also generating an estimated USD 300 billion in economic savings [6]. EBF alone has the potential to save more than one million children’s lives each year and substantially improve global health outcomes. Estimates suggest that between 1.3 and 1.45 million child deaths could be prevented annually with improved breastfeeding practices, particularly in low-income settings [7]. As a simple, cost-effective intervention, breastfeeding plays a vital role in enhancing infant survival and supports the achievement of child health targets outlined in the Millennium Development Goals [8].

In this broader context, Bahrain has made measurable progress in child health outcomes, with the infant mortality rate decreasing from 7.9 in 2010 to 5.8 per 1000 live births in 2020 [9]. However, despite national health policies that align with WHO recommendations, suboptimal EBF practices remain a concern. Cultural norms, hospital routines, and external influences—such as frequent family visits during hospitalization—may interfere with consistent breastfeeding practices. Furthermore, there is limited research evaluating how effectively these national breastfeeding initiatives are being implemented at the hospital level in Bahrain.

In many GCC countries, frequent hospital visits by extended family members may inadvertently disrupt early breastfeeding efforts, although this issue lacks empirical support in the current literature. More importantly, factors such as maternal age, parity, nationality, delivery method, anesthesia use, and previous breastfeeding experience have been consistently associated with EBF outcomes [10].

Delivery via cesarean section, particularly under general anesthesia, has been associated with delayed milk production and reduced breastfeeding rates when compared to vaginal births [11]. Institutional practices also play a critical role; hospitals that implement Baby-Friendly Hospital Initiative (BFHI) policies, such as early skin-to-skin contact and rooming-in, demonstrate higher breastfeeding success rates [12].

The onset of the COVID-19 pandemic introduced significant challenges to maternal and newborn care. Early recommendations, including mother–newborn separation in COVID-positive cases, disrupted routine breastfeeding support [13]. Bahrain adopted these policies, possibly impacting EBF during the pandemic. At the study hospital, these guidelines were implemented in March 2020, requiring temporary separation of mothers with suspected or confirmed COVID-19 until PCR results were available, along with visitor restrictions and modified breastfeeding counselling protocols. These measures likely influenced early initiation and continuation of breastfeeding during hospitalization. Despite these changes, no prior studies in Bahrain have quantitatively evaluated the impact of COVID-19 on breastfeeding behaviors. Therefore, this study aims to investigate the determinants of breastfeeding practices during hospitalization before and during the COVID-19 pandemic in Bahrain. This research will help inform policy development and targeted interventions to promote EBF, particularly during public health crises.

## 2. Materials and Methods

This study utilized a retrospective cohort to identify determinants of breastfeeding practices during hospitalization before and during the COVID-19 pandemic in Bahrain. Participants were grouped based on the timing of delivery: pre-pandemic group (March 2019–February 2020) and pandemic group (March 2020–March 2021, corresponding to the first year of the COVID-19 pandemic in Bahrain, during which the original SARS-CoV-2 strain was predominant). A simple random sampling method was used to select patient files from the register maintained at the selected hospital, utilizing the random number technique. This study was conducted at the selected tertiary hospital in Bahrain over a specified timeframe; data were obtained from the Hospital Information System (HOPE) and collected from March 2019 to March 2021. Participants included mothers who delivered and were discharged from the selected hospital during this period. Inclusion criteria consisted of all stable women who delivered at the selected hospital and stable infants with no contraindications to breastfeeding. To ensure the safety and appropriateness of breastfeeding for both mothers and infants, exclusions were applied. Mothers were excluded if they were HIV-positive in locations where artificial feeding was acceptable, currently using illicit substances (e.g., cocaine, heroin), or taking medications that could interrupt breastfeeding (e.g., radioactive isotopes, cancer chemotherapy). Additionally, mothers with active, untreated tuberculosis or active herpetic lesions on their breasts were excluded. Infants with galactosemia, dehydration, or weight loss exceeding 10% of birth weight were also excluded. These inclusion and exclusion criteria were implemented to ensure a representative sample and address potential confounding factors in investigating breastfeeding practices.

### 2.1. Sample Size

The total population over the two-year study period was approximately 4500 records. Based on this population size, the required sample size was calculated using a breastfeeding compliance rate of 35 ± 5%, with 95% confidence limits and a design effect of 1. The calculated minimum sample size was 325. To account for potential missing data or incomplete records, an additional 10% of files were reviewed, resulting in an initially targeted sample size of 360. However, after excluding records with missing or incomplete information, the final sample included in the analysis was 321. For the multivariable logistic regression analysis, multicollinearity among independent variables was assessed using the Variance Inflation Factor (VIF), with all variables showing VIF values below 2, indicating no significant multicollinearity. The model’s goodness of fit was evaluated using the Hosmer–Lemeshow test, which confirmed an adequate fit (*p* > 0.05).

### 2.2. Data Collection Procedures

Electronic medical records of postpartum women and their newborns discharged between March 2019 and March 2021 from a tertiary hospital were collected. The data were extracted from patient files and categorized into pre-pandemic and pandemic periods to examine changes in breastfeeding practices.

### 2.3. Measurements

Key maternal demographic characteristics, including age, nationality, gravidity, attendance at antenatal classes, mode of delivery, and type of anesthesia, were recorded. Infant-related variables included admission to other hospital units and reasons for mixed feeding, such as NICU admission, jaundice, or maternal preference. The primary outcome variable was breastfeeding practice, classified as EBF, mixed feeding, or formula feeding. The timeframe of each record was noted to determine its alignment with the pre-pandemic or pandemic period, allowing for a comparative analysis of breastfeeding trends before and during COVID-19. The structured data extraction ensured consistency and reliability in evaluating the impact of the COVID-19 pandemic, along with maternal demographic characteristics, on EBF practices during hospitalization.

### 2.4. Ethical Consideration

Ethical approval for this study was obtained from the Institutional Review Board of King Hamad University Hospital (Approval No. 22-545 on 17 October 2022). As a retrospective study, data were extracted from the electronic medical records of mothers and their newborns who had been discharged from the hospital. No direct interaction with participants was involved, and informed consent was waived by the IRB due to the anonymized nature of the data. To ensure confidentiality, all personal identifiers were removed, and access to patient records was limited to authorized researchers only. The study was conducted in accordance with the ethical principles outlined in the Declaration of Helsinki and institutional policies governing research on human subjects.

### 2.5. Statistical Analysis

The Statistical Package for the Social Sciences (SPSS version v23) was used to analyze the data. A summary of the dataset was provided via descriptive statistics. To investigate correlations between categorical variables, chi-square tests and Fisher’s exact tests for 2 × 2 tables were used. Monte Carlo adjustment was performed for low cell values.

Binary logistic regression analysis was performed to evaluate the predictive value of each variable. A *p*-value of <0.05 was considered statistically significant, while a *p*-value of <0.01 indicated a highly significant association. To determine the validity and resilience of the results, the proportion likelihood of error (*p*-value) and confidence intervals were considered while interpreting the data.

## 3. Results

### 3.1. Demographic and Clinical Characteristics of the Studied Mothers

Table 1 shows the demographic and clinical characteristics of the study groups. A total of 321 mothers were included and categorized into four groups according to feeding type and time period: pre-pandemic exclusive (*n* = 25), pre-pandemic mixed (*n* = 157), pandemic exclusive (*n* = 56), and pandemic mixed (*n* = 83). Maternal age was comparable across all groups, with the pandemic mixed group being slightly older on average (28.8 ± 5.4 years) and the pandemic exclusive group slightly younger (28.0 ± 5.5 years). Most mothers were multiparous, particularly in the pandemic mixed group (88%), while the proportion of primiparous mothers ranged from 12% in the pandemic mixed group to 28.6% in the pandemic exclusive group. The distribution of Bahraini and non-Bahraini mothers was relatively even. Only a few mothers were admitted to another unit during their stay (8.3% in the pre-pandemic mixed group and 6.5% in the pandemic mixed group), while no admissions occurred in either of the exclusive breastfeeding groups.

Gestational age at delivery was also similar across groups, ranging between 38.9 and 39.2 weeks. Differences in mode of delivery were observed between feeding groups. Vaginal deliveries, including Normal Spontaneous Vaginal Delivery (NSVD) and Spontaneous Vaginal Delivery (SVD), were more frequent among mothers who practiced exclusive breastfeeding. In contrast, Caesarean sections, both Elective Caesarean Section (ELCS) and Emergency Caesarean Section (EMCS), appeared more often among mothers in the mixed-feeding groups. For example, almost all mothers in the pre-pandemic exclusive group delivered by NSVD (92%), while the pandemic exclusive group had a predominance of SVD (55.4%). In contrast, the pandemic mixed group showed the lowest rate of NSVD (13.3%), with higher proportions of EMCS (32.5%) and SVD (32.5%).

Patterns of anesthesia use also varied. In the pre-pandemic exclusive group, Epidural Anesthesia (EA) and Spinal Anesthesia (SA) were equally common (50% each). Spinal Anesthesia was the most frequently used technique in the pre-pandemic mixed (41%) and pandemic mixed (53.8%) groups, whereas Epidural Anesthesia was more common in the pandemic exclusive group (44.4%). General Anesthesia (GA) and No Anesthesia (NA) were used less frequently across all groups.

### 3.2. Reasons for Infant Feeding Practices

Table 2 shows the main reasons for infant feeding practices. Before the pandemic, mixed feeding was most commonly due to maternal request (43.9%) and mothers remaining in the recovery room (32.5%), with less frequent reasons including labor and delivery (5.7%), low blood sugar (5.1%), jaundice (2.5%), medical indications (1.9%), ICU admission (1.9%), and inadequate milk output (1.2%). During the pandemic, maternal request (39.0%) and recovery room stay (32.5%) remained the leading causes, while pending COVID-19 test results appeared in 10.4% of cases. Other less common reasons included labor and delivery (3.9%), low blood sugar (2.6%), medical indications (2.6%), jaundice (1.3%), and NICU transfer (1.3%).

For exclusive breastfeeding, pre-pandemic cases were mostly influenced by mothers in the recovery room (60.0%), labor and delivery (16.0%), maternal request (16.0%), and NICU transfer (8.0%). During the pandemic, exclusive breastfeeding occurred mainly when mothers were in labor and delivery (16.0%) or the newborn was transferred from the NICU (8.0%).

### 3.3. Breastfeeding Practices

**Figure 1:**
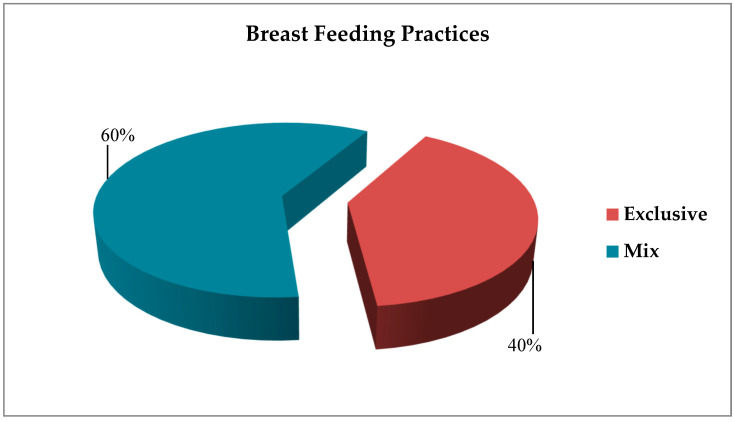
Breastfeeding practices of mothers in the pre-pandemic period (*n* = 182).

**Figure 2:**
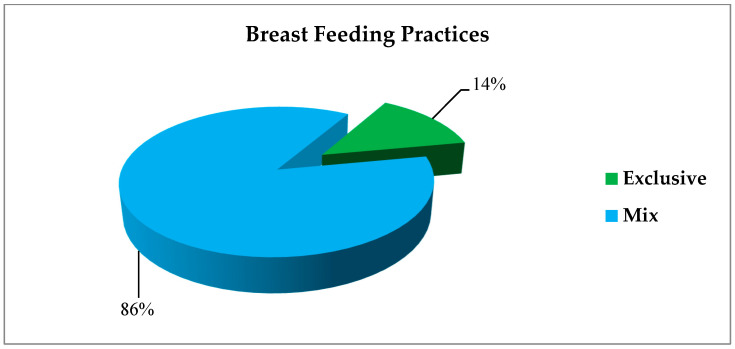
Breastfeeding practices of mothers in the pandemic period (*n* = 139).

During the pre-pandemic period (*n* = 182), 40% of mothers opted for EBF, while the majority (60%) practiced mixed feeding (Figure 1). In the pandemic period (*n* = 139), 14% of mothers exclusively breastfed their infants, while 86% practiced mixed feeding (Figure 2).

### 3.4. Relation Between Demographic Characteristics and Breastfeeding Practices

In the pre-pandemic period, the mode of delivery showed a statistically significant relationship with breastfeeding practice (χ^2^ = 18.93, *p* < 0.001), indicating that delivery method influenced whether a mother practiced exclusive or mixed feeding. Other demographic variables—including maternal age, parity status, nationality, gestational age, and admission to another unit—were not significantly associated with breastfeeding method.

During the pandemic period, two variables showed significant associations. Parity status was significantly related to feeding practice (χ^2^ = 6.17, *p* = 0.016), with multiparous women more likely to practice EBF. As in the pre-pandemic period, mode of delivery remained a strong predictor (χ^2^ = 37.06, *p* < 0.001). Other variables, such as maternal age, nationality, and gestational age, did not demonstrate significant associations with feeding choice during the pandemic (Table 3).

### 3.5. Predictive Factors of Breastfeeding Practice (Pre-Pandemic and Pandemic)

Table 4 presents the results of the multivariate logistic regression analysis used to identify predictors of breastfeeding practices before and during the pandemic. The findings show that the mode of delivery was the only variable that significantly influenced breastfeeding. Mothers who delivered vaginally either by NSVD or SVD were more likely to exclusively breastfeed, whereas caesarean deliveries, both ELCS and EMCS, were associated with mixed feeding.

Before the pandemic, women who had a vaginal delivery were nearly four times more likely to exclusively breastfeed compared with those who underwent Caesarean section (Exp(B) = 3.696, 95% CI: 1.602–8.530, *p* = 0.002). During the pandemic, this relationship remained significant, although the strength of association was lower; vaginal delivery was linked to more than double the likelihood of exclusive breastfeeding (Exp(B) = 2.238, 95% CI: 1.483–3.378, *p* < 0.001). Other maternal factors, including age, parity, nationality, and gestational age, did not show any significant association with breastfeeding practices in either period (all *p* > 0.05). Table 4 shows predictive factors of breastfeeding practice pre-pandemic and during the pandemic.

## 4. Discussion

This study explored the influence of the COVID-19 pandemic, delivery method, and maternal characteristics on breastfeeding practices during hospitalization in Bahrain. Findings revealed a decline in EBF during the pandemic, with a corresponding rise in mixed feeding. Vaginal delivery and prior maternal experience (multiparity) were strongly associated with higher EBF rates, while cesarean birth and primiparity emerged as key risk factors for early supplementation.

EBF declined during the pandemic period in our sample, while mixed feeding became more common. These shifts are concerning in a context where community-level EBF is already suboptimal across parts of the Eastern Mediterranean region, suggesting that even short in-hospital disruptions may have downstream effects on post-discharge feeding trajectories [14,15,16].

Mode of delivery emerged as a central influence. Mothers who experienced vaginal birth were more likely to sustain EBF during hospitalization than those who had a cesarean birth. This aligns with broader literature showing that cesarean delivery can delay breastfeeding initiation, reduce the likelihood of establishing effective latch, and contribute to early supplementation pathways that include postoperative discomfort, maternal-infant separation, and variation in perioperative practices such as skin-to-skin contact [17,18,19].

Parity also mattered particularly under pandemic conditions. Multiparous mothers generally maintained breastfeeding more effectively than primiparous mothers, a pattern consistent with regional reports that prior experience, confidence, and family encouragement support breastfeeding continuation in Middle Eastern and Gulf populations [20,21].

Support needs around the time of birth may help explain some of these differences. Evidence indicates that women recovering from operative birth, as well as first-time mothers, often require more hands-on guidance to establish positioning and latch; when that support is insufficient, early supplementation is more likely [22].

Pandemic-related hospital policies likely contributed to the feeding shifts we observed. International and U.S. data collected during COVID-19 describe changes in postpartum care routines, variable mother-newborn contact while infection status was clarified and altered staff workflows—all factors that can interrupt early breastfeeding opportunities [23,24]. Accounts from mothers and clinicians in pandemic-affected settings also describe how visitation limits reshaped the immediate postpartum environment: quieter rooms sometimes aided rest, yet reduced access to trusted support persons could hinder practical breastfeeding help. Qualitative work from Spain captures this tension, with families reporting both bonding benefits and support challenges under visitation restrictions [25].

Cultural context is critical in interpreting our findings. In many Gulf and broader regional communities, early postpartum recovery is traditionally supported by close female relatives and other family members who assist directly with infant care and feeding. Restrictions that limited these companions during the pandemic may, therefore, have had a disproportionate impact on breastfeeding establishment in our setting. Regional reviews underscore the importance of culturally grounded family support in sustaining breastfeeding [20].

Patterns in the reasons documented for mixed feeding offer additional insight. Maternal request and temporary unavailability (for example, while recovering after birth) were common in both time periods; during the pandemic, awaiting maternal COVID-19 results emerged as an added barrier, illustrating how necessary infection control processes can inadvertently separate mothers and infants at a critical time for lactation [13]. Guidance developed during the pandemic emphasized maintaining breastfeeding and human milk use whenever safely possible, even when testing or isolation protocols were in place [24].

Differences in anesthesia exposure across groups may have influenced early contact opportunities and maternal readiness to feed. Literature comparing aesthetic approaches around cesarean birth suggests that strategies minimizing maternal sedation and facilitating earlier interaction can support breastfeeding success [17,19,22].

Across analyses, birth mode remained the most consistent predictor of feeding pattern in our cohort, even after accounting for maternal characteristics and pandemic-related variables. These findings reinforce the need for structured, proactive lactation pathways for mothers who deliver by cesarean, particularly when health system stressors (such as pandemic precautions) heighten the risk of mother-infant separation. Elements may include immediate or skin-to-skin when feasible, timely initiation of milk expression if separation occurs, targeted pain management that enables positioning, and intensified bedside coaching once the mother is able to participate. Evidence across multiple settings suggests that without such measures, cesarean birth can exert lasting negative effects on breastfeeding exclusivity and duration [17,18,19,22].

In summary, our data suggest that pandemic-related operational changes layered onto existing regional breastfeeding challenges, amplifying the shift toward mixed feeding during hospitalization. Yet, the core drivers of EBF remained familiar: birth mode and maternal experience. Policies that preserve maternal-infant contact, integrate culturally important family support within infection control frameworks, and post-cesarean lactation care, especially for first-time and younger mothers, may help protect breastfeeding during future public health emergencies [14,15,20,24].

The data collection method from electronic patients’ files is limited, so factors like the social and financial status of mothers were not measured in the current study, even though from the literature reviewed, they are one of the factors that affect breastfeeding practice. In addition, the system is not getting updated in regard to the patient’s educational level and workplace, for example.

### 4.1. Recommendations

#### 4.1.1. Nursing Care

The decline in EBF rates during the pandemic highlights the critical role of nurses in promoting and supporting breastfeeding, especially during times of crisis. Moving forward, nursing care must prioritize accessible, evidence-based breastfeeding education and support across all care settings—including virtual platforms. Nurses should be trained to recognize the unique challenges faced by younger, first-time, and post-cesarean mothers, tailoring interventions accordingly. Strengthening postpartum follow-up and lactation consulting services can help close gaps in care and sustain EBF rates beyond the immediate postnatal period.

#### 4.1.2. Patient Health

EBF is essential for infant health, providing optimal nutrition and immune protection. The observed decrease during the pandemic may have long-term health implications for children born in that period. Public health strategies must target high-risk groups—such as primiparous women and those undergoing cesarean sections—to ensure that they receive targeted education and support. Enhancing maternal confidence and self-efficacy through peer support groups and community programs can improve breastfeeding outcomes and, consequently, overall child health.

#### 4.1.3. Research

These findings underscore the need for ongoing research into the factors that influence breastfeeding behaviors, particularly in emergency contexts like pandemics. Future research should explore the barriers faced by different maternal demographics and the effectiveness of interventions tailored to age, social and financial status, parity, and mode of delivery. Longitudinal and mixed-methods studies could also help determine the lasting impacts of disrupted breastfeeding practices on child development and maternal well-being. Additionally, investigating the role of digital health tools and tele-lactation services could inform more resilient models of care in future public health emergencies. Supplementing electronic data with surveys or interviews can provide a more comprehensive understanding of maternal needs.

## 5. Conclusions

The study highlighted the factors that influence breastfeeding practices among mothers both before and during the pandemic. The analysis revealed significant associations between EBF rates and delivery methods, with mothers who underwent cesarean delivery exhibiting lower rates of EBF compared with those who had other modes of delivery.

These findings highlight the crucial role of delivery methods in promoting EBF practices, especially amid the pandemic. Although maternal age and gravidity were linked to EBF rates, factors like nationality, attendance at prenatal classes, and gestational age did not show significant correlations. This suggests that delivery methods may be a more influential determinant of breastfeeding practices compared to other demographic factors.

## Figures and Tables

**Table 1:** Percentage distribution of the sample studied according to their demographic characteristics (*n* = 321).

Variable	Pre-Exclusive (*n* = 25)	Pre-Mixed (*n* = 157)	Pandemic Exclusive (*n* = 56)	Pandemic Mixed (*n* = 83)
Maternal Age				
Mean ± SD	28.35 ± 6.09	28.35 ± 6.09	27.98 ± 5.46	28.81 ± 5.40
Parity (%)				
Multipara	72.00%	79.50%	71.40%	88.00%
Primipara	28.00%	20.50%	28.60%	12.00%
Nationality (%)				
Bahraini	52.00%	40.40%	48.20%	50.60%
Non-Bahraini	48.00%	59.60%	51.80%	49.40%
Admitted to Another Unit (%)	0.00%	8.30%	0.00%	6.50%
Gestational Age (weeks) Mean ± SD	39.07 ± 1.30	38.93 ± 1.32	39.17 ± 1.35	38.90 ± 1.53
Mode of Delivery				
NSVD	92.00%	46.20%	35.70%	13.30%
SVD	4.00%	3.80%	55.40%	32.50%
ELCS	4.00%	25.00%	5.40%	21.90%
EMCS	0.00%	25.00%	3.20%	32.50%
Type of Anesthesia				
EA	50.0% (*n* = 2)	18.1% (*n* = 105)	44.4% (*n* = 9)	15.4% (*n* = 52)
SA	50.0% (*n* = 2)	41.0% (*n* = 105)	33.3% (*n* = 9)	53.8% (*n* = 52)
GA	—	25.7% (*n* = 105)	—	25.0% (*n* = 52)
NA	—	15.2% (*n* = 105)	22.2% (*n* = 9)	5.8% (*n* = 52)

NSVD: Normal Spontaneous Vaginal Delivery (vaginal delivery without complications or interventions), SVD: Spontaneous Vaginal Delivery vaginal delivery (that is spontaneous but may include minor interventions such as labor augmentation or episiotomy), ELCS: Elective Cesarean Section, EMCS: Emergency Cesarean Section, EA: Epidural Anesthesia, SA: Spinal Anesthesia, GA: General Anesthesia, NA: No Anesthesia.

**Table 2:** Reasons for infant feeding practices (pre- and during pandemic periods).

Reason	Pre-Pandemic Mixed (*n* = 157)	Pandemic Mixed (*n* = 83)	Pre-Pandemic Exclusive (*n* = 25)	Pandemic Exclusive (*n* = 56)
Inadequate Output	1.20%	—	—	—
Jaundice	2.50%	1.30%	—	—
Low Blood Sugar	5.10%	2.60%	—	—
Medically Indicated	1.90%	2.60%	—	—
Mother in ICU	1.90%	—	—	—
Mother in Labor & Delivery (LD)	5.70%	3.90%	16.00%	16.00%
Mother in Recovery Room (RR)	32.50%	32.50%	60.00%	—
Mother’s Request	43.90%	39.00%	16.00%	—
Transferred from NICU	—	1.30%	8.00%	8.00%
COVID-19 Test Pending	—	10.40%	—	—

**Table 3:** Correlation between demographic characteristics and breastfeeding practice (pre- and during pandemic periods).

Variable	Pre-Pandemic χ^2^ (*p* Value)	Pandemic χ^2^ (*p* Value)
Mother’s Age	6.43 (0.09) ^mc^	2.08 (0.572) ^mc^
Parity	0.743 (0.389)	6.17 (0.016 *)
Nationality	1.12 (0.290)	0.119 (0.863) ^$^
Admitted to Another Unit	2.22 (0.135)	—
Gestational Age	0.488 (0.625) ^#^	1.08 (0.0281) ^#^
Mode of Delivery	18.93 (<0.001 *) ^mc^	37.06 (<0.001 *) ^mc^

mc: Monte Carlo correction for Chi-square test **#**: Independent *t*-test. $: Fisher’s exact test. *: Statistically significant at *p* < 0.05.

**Table 4:** Predictive factors of breastfeeding practice (pre-pandemic and pandemic).

Variables	B (Pre-Pandemic)	Exp(B) (95% CI) Pre-Pandemic	Sig. Pre-Pandemic	B (Pandemic)	Exp(B) (95% CI) Pandemic	Sig. Pandemic
Age	−0.038	0.962 (0.574–1.615)	0.885	0.195	1.216 (0.716–2.063)	0.470
Parity	−0.675	0.509 (0.203–1.277)	0.150	0.262	1.300 (0.590–2.865)	0.516
Nationality	−0.120	0.887 (0.309–2.547)	0.824	−0.814	0.443 (0.164–1.196)	0.108
Delivery	1.307	3.696 (1.602–8.530)	0.002 *	0.806	2.238 (1.483–3.378)	<0.001 *
Gestational age	−0.002	0.998 (0.526–1.894)	0.995	0.184	1.203 (0.734–1.970)	0.464

B: Regression coefficient. Sig.: Significance (*p* value). Exp(B): Odds ratio. CI: Confidence interval. *: Statistically significant at *p* < 0.05.

## Data Availability

The original contributions presented in this study are included in the article. Further inquiries can be directed to the corresponding author.
